# Deviant Peer Affiliation and Adolescent Tobacco and Alcohol Use: The Roles of Tobacco and Alcohol Information Exposure on Social Networking Sites and Digital Literacy

**DOI:** 10.3390/bs12120478

**Published:** 2022-11-25

**Authors:** Xuqing Bai, Liangshuang Yao, Changying Duan, Xiaojun Sun, Gengfeng Niu

**Affiliations:** 1 School of Psychology, Central China Normal University, Wuhan 430079, China; 2 Key Laboratory of Adolescent Cyberpsychology and Behavior (CCNU), Ministry of Education, Wuhan 430079, China

**Keywords:** deviant peer affiliation, SNS, tobacco and alcohol information, tobacco and alcohol use, digital literacy, adolescents

## Abstract

Due to the prevalence and severe harm of adolescent tobacco and alcohol use, researchers have been paying attention to its influencing factors. From the perspective of the ecological techno-subsystem theory, this study aimed to examine the correlations between deviant peer affiliation, tobacco and alcohol information exposure on social networking sites (SNSs), and adolescent tobacco and alcohol use, as well as the potential protective role of digital literacy. In total, 725 adolescents were recruited to participate in this study. The results showed that deviant peer affiliation was positively associated with adolescent tobacco and alcohol use, SNS tobacco and alcohol information exposure mediated this association, and digital literacy moderated the relationship between SNS information exposure and tobacco and alcohol use. Specifically, the association between SNS tobacco and alcohol information exposure and adolescent tobacco and alcohol use was weaker for those with high digital literacy. These findings not only explore the combined effects of offline and online risk factors but also provide guides for preventing adolescents’ tobacco and alcohol use by cultivating and enhancing digital literacy.

## 1. Introduction

Tobacco and alcohol use are prevalent social problems worldwide, especially among adolescents [1]. It may cause great harm to adolescents’ health and adaptation—tobacco and alcohol use cause brain damage [2], poor academic performance [3,4], and aggressive behaviors [5] among adolescents. Therefore, its influencing factors have been the focus of researchers, among which peer relationship, especially deviant peer affiliation, has received attention [6,7,8,9]. This is because the influence of peer relationships surpasses other interpersonal relationships (such as parent–child relationships) and becomes one of the main factors affecting adolescents’ psychological and behavioral development [10].

At the same time, in the current information society, various digital technologies and applications (such as mobile phones and SNSs) have become prominent factors influencing adolescents’ problem behaviors [11,12]. In particular, SNSs are of great appeal and popular for adolescents [13], providing conveniences for them to obtain information and communicate. Due to the features of SNSs (such as freedom of content and lack of regulation), there may exist bad information, such as tobacco and alcohol information posted by other users [11,14,15], which may have negative influences on adolescents. Meanwhile, SNSs are the main platforms for online peer communication [16]. Online peer communication on SNSs may be associated with offline peer communication. From the perspective of the ecological techno-subsystem theory [17], ecosystems, represented by peers, and technology systems, represented by SNSs, will jointly and interactively work on the development of adolescents. In addition, digital literacy is an important factor influencing individuals’ adaptation to the current information society. In particular, it may prevent the negative influences of modern technology (such as negative information exposure on SNS) [18,19,20]. Based on these, under the perspective of the ecological techno-subsystem theory [17], this study aimed to examine the mechanism underlying the association between deviant peer affiliation and adolescent tobacco and alcohol use, focusing on the mediating role of SNS tobacco and alcohol information exposure and the moderating role of digital literacy.

### 1.1. Deviant Peer Affiliation and Adolescent Tobacco and Alcohol Use

Adolescent self-awareness occurs in the stage of adolescents’ independent development, during which they gradually separate from their parents in emotion, behavior, and opinion [21]. At the same time, adolescents are interacting more with their peers, who have a greater impact on them than their parents [10]. Different peer factors and relationships may have different effects on adolescents, among which deviant peer affiliation may have a negative effect [6]. Deviant adolescents exhibit a high-incidence group of problem behaviors (such as smoking, drinking, skipping classes, etc.) [22], and associating with them may make adolescents more susceptible to problem behaviors such as tobacco and alcohol use.

Based on the social learning theory [23], adolescents may imitate the behaviors of deviant peers, such as smoking and drinking [24]. Further, adolescents also learn the outcomes of deviant peer alcohol and tobacco use [25]. For example, smoking and drinking are considered cool and anxiety-relieving behaviors by adolescents [26]. This erroneous feedback may reinforce adolescents’ alcohol and tobacco use [25,26]. Second, deviant peers may use alcohol and tobacco together as a way of maintaining friendships. Adolescents may mingle with deviant peers through alcohol and tobacco use. Specifically, adolescents who originally did not smoke or drink alcohol may use tobacco and alcohol to close the gap and enhance their relationships with their deviant peers as a result of their interactions with them [27]. Furthermore, extensive evidence showed that deviant peer affiliation can affect alcohol and tobacco use among adolescents [6,7,8,9]. Thus, we hypothesized that deviant peer affiliation is positively associated with adolescent tobacco and alcohol use (Hypothesis 1).

### 1.2. The Mediating Effect of Tobacco and Alcohol Information Exposure on Social Networking Sites

In the current information society, the influence of Internet factors such as Internet information and social networking sites on adolescents cannot be ignored. SNSs, an extension of offline peer interaction with adolescents in cyberspace [16], are important platforms for youth peer interaction and self-expression [28]. Specifically, there is some overlap between the interaction of adolescents on SNSs and offline [29]. At the same time, SNSs are platforms for self-expression, and deviant adolescents post information about tobacco and alcohol use on SNSs, such as drinking together in bars and sharing tobacco together [26]. As a result, adolescents who associate with deviant peers may receive information about tobacco and alcohol use posted by deviant peers [19,30]. In addition to being exposed to alcohol and tobacco information posted by their deviant peers, adolescents are also exposed to alcohol and tobacco information promoted on SNS platforms [12]. According to the peer cluster theory [31], due to the interaction with deviant peers, adolescents may be more positive and less avoidant of messages about tobacco and alcohol use and, therefore, will be more passively exposed to them [32]. Furthermore, adolescents will actively browse and respond to SNS tobacco and alcohol information. This is to express that they have the same attitude as their deviant peers [33] and to fit into the group of their deviant peers [33]. Empirical research showed that deviant peers are associated with SNS tobacco and alcohol information exposure [9,26,34]. 

In addition, SNS tobacco and alcohol information exposure may be related to adolescent alcohol and tobacco use. Due to the lack of platform regulation, there is huge tobacco- and alcohol-related information on SNSs, including the information posted by people, some advertisements, etc. [14]. 

Drawing on the cultivation theory [35], the mainstream information and concepts disseminated by the media (including SNSs) will subconsciously influence individuals. First, the influence of the media is unconscious [35]. One study found that when an individual is exposed to SNS information multiple times, the illusory truth effect will occur, that is, a subconscious belief in that information [36]. Individual behavior is also subtly influenced by SNS information [37]. As a result, SNS tobacco and alcohol information may be subconsciously related to adolescents’ tobacco and alcohol use. Second, the information disseminated by the media is considered to be the mainstream social norm [35]. Even the descriptions of tobacco and alcohol information on SNSs are mostly positive; for example, e-cigarettes are harmless, and drinking alcohol reduces stress [19,38]. Positive descriptions may convey to adolescents that alcohol and tobacco use conform to social norms, thereby further influencing their tobacco and alcohol use. Studies have also shown that SNS tobacco and alcohol information exposure increases the normative perceptions of tobacco and alcohol use and affects tobacco and alcohol use [11,24,39]. Moreover, the results of longitudinal studies and meta-studies showed that SNS tobacco and alcohol information exposure will lead to more tobacco and alcohol use [15,40,41]. Thus, we hypothesize that SNS tobacco and alcohol information exposure mediates the relationship between deviant peer affiliation and adolescent tobacco and alcohol use (Hypothesis 2).

### 1.3. The Moderating Effect of Digital Literacy

In addition, the potential protective factors against tobacco and alcohol use have been given more and more importance [42], which is of great theoretical and practical significance. Previous studies have mainly examined the positive factors that reduce the risk of tobacco and alcohol use from the perspective of microsystems, such as parental [43] and school education [26]. However, in the current information society, the technology system is also one of the important systems to be explored. According to the message interpretation process (MIP) model [44], individuals’ logical or rational thinking about digital information influences their subsequent behavior [45], and such logical or rational thinking is the reflection of digital literacy in the technology system. 

Digital literacy is the ability of individuals to explain and respond to digital information, including their ability to identify and think critically about digital information [18]. Relevant empirical studies have shown that digital literacy can reduce the negative impact of negative information exposure (e.g., junk food) [46] and problematic behaviors (e.g., gaming addiction and cyberbullying) [47,48]. In that way, digital literacy may be a moderating factor of SNS tobacco and alcohol information exposure on tobacco and alcohol use. Specifically, adolescents with higher digital literacy ability will critically think about tobacco and alcohol information on SNSs and are less influenced by SNS tobacco and alcohol use information. By contrast, adolescents with low digital literacy have less critical thinking and are more susceptible to the positive descriptions of alcohol and tobacco information [49], resulting in tobacco and alcohol use behaviors [30]. In addition, digital-literacy-related variables such as social media literacy and media literacy can have a moderating effect between online risk information and problematic behaviors [20,50]. For example, individuals with high digital literacy realize the positive image deliberately created by advertising companies for sales [49], mitigate the negative impact of SNS ideal photos on individual body satisfaction [20], and reduce the impact of the media on individual junk food intake behavior [46]. Moreover, intervention studies show that digital literacy training can reduce the impact of misleading information on adolescents and make reasonable and correct behaviors [19]. Therefore, digital literacy may become a moderating factor in SNS tobacco and alcohol information exposure and tobacco and alcohol use (Hypothesis 3).

### 1.4. The Present Study

**Figure 1:**
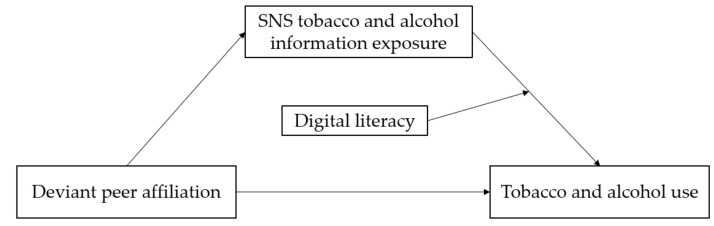
The constructed moderated mediating model.

Considering the severe harm of tobacco and alcohol use and the increase in SNS tobacco and alcohol information, under the perspective of the ecological techno-subsystem theory and online and offline interaction, this study constructed a moderated mediating model (see Figure 1) to examine the mechanism underlying the relationship between deviant peer affiliation and adolescent tobacco and alcohol use in the current information society, the mediating effect of SNS tobacco and alcohol information exposure, and the moderating effect of digital literacy.

## 2. Materials and Methods

### 2.1. Participants

Participants were recruited from middle and high schools in different regions of China and participated in the experiment through a paper version of the questionnaire. A total of 812 middle school students voluntarily participated in our study, among whom 725 provided valid questionnaires (excluding the cases with missing and regular data), and the percentage of people who completed the entire survey was 89.29%. Across the remaining sample, 387 participants were male, and 337 were female. The average age of the participants was 15.390 years old (*SD* = 1.641).

### 2.2. Procedure

The research design was approved by the institutional ethical committee of the authors’ university. After obtaining the permission of the headteacher and the guardian, the psychology department personnel who have received professional training randomly entered different classes and explained the experiment to the students. After giving informed consent and voluntarily participating in the experiment, the subjects filled in the questionnaire anonymously according to their real situation.

### 2.3. Measurements

#### 2.3.1. Deviant Peer Affiliation

The Chinese version of the deviant peer affiliation scale [51] was used to measure the deviant behavior of adolescent peers (e.g., smoking, drinking, skipping classes, etc.). The participants were asked to respond on a five-point Likert scale (1 = none, 2 = rarely, 3 = some, 4 = most, 5 = all) on each of the eight items. The higher the score, the more peer interaction on the track. In this study, Cronbach’s alpha for this scale was 0.847.

#### 2.3.2. Tobacco and Alcohol Use

The Chinese version of the tobacco and alcohol use scale [52] was adopted to measure the frequency (2 items) and amount (2 items) of alcohol and tobacco use among adolescents in the past 30 days, which is a widely used measurement worldwide. For the frequency, the participants were asked to respond to the frequency of tobacco and alcohol use on a 6-point Likert-type scale ranging from 1 (never smoking/drinking) to 6 (smoking/drinking for 20–30 days), (e.g., “How many days have you been smoking in the past 30 days?”); for the amount, the participants were asked to respond to the amount of tobacco and alcohol use on a 6-point Likert-type scale ranging from 1 (never smoking/drinking) to 6 (10 cigarettes/10 glasses of alcohol and above) (e.g., “In the past 30 days, on the days you smoked, about how many cigarettes do you smoke a day?”). The responses were averaged to form a measure of the adolescents’ tobacco and alcohol use, with higher scores indicating more tobacco and alcohol use. In this study, Cronbach’s alpha for this scale was 0.800.

#### 2.3.3. SNS Tobacco and Alcohol Information Exposure

The Chinese version of the SNS exposure scale [43] was used to measure the frequency of individuals’ exposure to tobacco and alcohol information on social networking sites, including WeChat, Weibo, and Q-zone (e.g., “I saw alcohol information on Weibo”). The participants rated the frequency of tobacco and alcohol information exposure on social networking sites (1 = never; 5 = always). Higher scores indicated more frequency of smoking and alcohol messages on social networking sites. In this study, Cronbach’s alpha for this scale was 0.908.

#### 2.3.4. Digital Literacy

The digital literacy scale [18] was used to measure the digital literacy level of adolescents, which includes twenty items and could be divided into six dimensions: photo-visual literacy, replication literacy, information literacy, branching literacy, social–emotional literacy, and real-time reasoning literacy (e.g., “Ignoring ads that pop up while looking for information for an assignment”). The participants were asked to respond to twenty items on a five-point scale ranging from 1 (very difficult) to 5 (very easy). The higher the score, the higher the digital literacy. In this study, the digital literacy scale was translated into Chinese through standard procedures. Good validity and reliability were observed with this Chinese version scale, and confirmatory factor analysis indicated an acceptable fit: *χ^2^/df* = 2.992, RMSEA = 0.052, CFI = 0.925, CFI = 0.907, SRMR = 0.049; Cronbach’s alpha for the six dimensions were 0.829, 0.763, 0.796, 0.719, 0.753 and 0.406, respectively; Cronbach’s alpha for this scale was 0.886.

## 3. Results

### 3.1. Descriptive and Correlational Analysis

Table 1 shows the descriptive statistics for, and the intercorrelations among, the study variables. Deviant peer affiliation, tobacco and alcohol use, and SNS tobacco and alcohol information exposure were positively correlated with each other. Digital literacy was negatively correlated with tobacco and alcohol use.

### 3.2. Testing for the Proposed Moderated Mediation Model

The PROCESS macro (Model 14) in SPSS was further used to test the mediating effect of SNS tobacco and alcohol information exposure on deviant peer affiliation and tobacco and alcohol use and the moderating effect of digital literacy in the latter half of the mediation model. The results showed (Table 2) that deviant peer affiliation was significantly positively associated with tobacco and alcohol use (*B* = 0.187, *p* < 0.001), and SNS tobacco and alcohol information exposure (*B* = 0.464, *p* < 0.001). SNS tobacco and alcohol information exposure was also significantly positively associated with tobacco and alcohol use (*B* = 0.080, *p* < 0.01). The results indicated that SNS tobacco and alcohol information exposure mediated the relationship between deviant peer affiliation and tobacco and alcohol use among adolescents. Therefore, Hypothesis 2 was supported.

**Figure 2:**
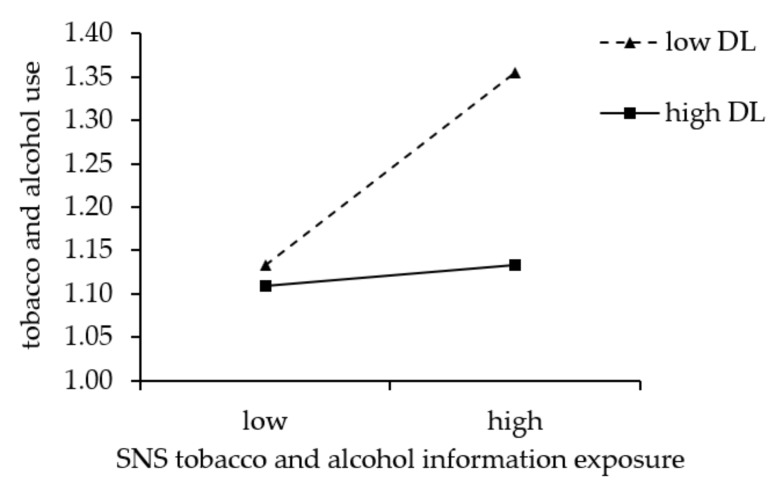
Moderating effect of digital literacy (DL) on the relationship between SNS tobacco and alcohol information exposure and tobacco and alcohol use.

The interaction between SNS tobacco and alcohol information exposure and digital literacy on adolescent tobacco and alcohol use was significant (*B* = −0.119, *p* < 0.01). To more clearly describe the moderating effect of digital literacy (see Figure 2), a simple slope plot was drawn for digital literacy at mean minus one standard deviation (low digital literacy) and mean plus one standard deviation (high digital literacy). Through group regression analysis, it was found that with the increase in digital literacy, the predictive effect of SNS tobacco and alcohol information exposure on tobacco and alcohol use gradually weakened (from *B_simple_* = 0.145, *p* < 0.001, weakened to *B_simple_* = 0.016, *p* = 0.601). Additionally, when digital literacy was *M*-*1SD*, M, and *M+1SD*, the mediating effect size of SNS tobacco and alcohol information exposure and their 95% confidence interval were 0.067 [0.012, 0.129] and 0.007 [−0.016, 0.033], indicating that with the improvement in the level of individual digital literacy, the mediating effect of SNS tobacco and alcohol information exposure on deviant peer affiliation and tobacco and alcohol use gradually weakened. Thus, Hypothesis 3 was supported.

## 4. Discussion

From the perspective of the ecological techno-subsystem theory, and the real lives of adolescents, this study was designed to examine the mediating role of SNS tobacco and alcohol information exposure in deviant peer affiliation and tobacco and alcohol use and the protective role of digital literacy. The results revealed that deviant peer affiliation was positively associated with tobacco and alcohol use through the mediating effect of SNS tobacco and alcohol information exposure. The results also revealed that digital literacy moderated the effects of SNS tobacco and alcohol information exposure on tobacco and alcohol use. Specifically, the association between SNS tobacco and alcohol information exposure and tobacco and alcohol use was weaker for adolescents with higher digital literacy. These findings expand previous studies, as this study not only provides a deeper understanding of the combined influence of offline and online factors on adolescents, namely the impact of deviant peer association and SNS tobacco and alcohol message exposure on adolescent tobacco and alcohol use, but it also sheds light on digital literacy as a protective factor against tobacco and alcohol use among adolescents in the information society.

### 4.1. Deviant Peer Affiliation and Adolescent Tobacco and Alcohol Use

First, consistent with previous studies [6,7,9], deviant peer affiliation was positively correlated with alcohol and tobacco use in adolescents. Adolescents are in a critical period of the development of self-identity. An important factor in the formation of adolescents’ self-identity is knowing who they are and how they should develop their identity by communicating with their peers [53]. Peers have a huge physical and mental impact on adolescents. Adolescents internalize the problem behaviors of deviant peers and incorporate them into their own identity, thus adopting problem behaviors such as alcohol and tobacco use [54]. At the same time, adolescents who are unable to integrate into a group may be subject to social exclusion [55] and bullying [56], when peer relationships are extremely important. Therefore, adolescents will imitate the use of tobacco and alcohol to integrate into a group of deviant peers, especially in the Eastern collectivist culture [57].

### 4.2. SNS Tobacco and Alcohol Information Exposure as a Mediator

The results further revealed the inner mediating mechanism of exposure to SNS tobacco and alcohol information in the current information society, finding that SNS tobacco and alcohol information exposure plays a mediating role between deviant peer affiliation and adolescent tobacco and alcohol use. Consistent with the ecological techno-subsystem theory, there is a relationship between ecological systems and technical systems. In this study, deviant peer affiliation was also associated with SNS tobacco and alcohol information exposure. Digitally native youth groups are the main recipients and feedbackers of SNS information. From the perspective of recipients, SNSs are platforms for adolescents to show themselves and interact with peers. Deviant peers post photos of smoking and drinking on media platforms, and adolescents are exposed to such information about alcohol and tobacco [38,58]. This reflects the overlap between adolescents’ offline and online communication, that is, SNSs are the extension of adolescents’ offline communication in cyberspace [16]. From the perspective of feedbackers, unlike the one-way output of tobacco and alcohol information in traditional media [19], adolescents also have two-way interactions with tobacco and alcohol information on SNSs [30,33], through features such as likes, comments, and reposts. This further affects the clustering algorithm and personalization algorithm of SNSs to recommend tobacco and alcohol information for adolescents [12]. 

In addition, as discussed, there may be various negative information on SNSs, such as pornography, violence, alcohol, and tobacco. Adolescents who are active in SNSs have a higher chance of being exposed to negative information and developing problematic behaviors [30]. This study found that SNS tobacco and alcohol information exposure was positively associated with adolescent tobacco and alcohol use, which is consistent with the ecological techno-subsystem theory: Technology systems act on adolescent development. Similar to the tobacco and alcohol information in traditional media [30], SNS tobacco and alcohol information conveys the cognition that the use of tobacco and alcohol conforms to social norms [39], leading to the use of tobacco and alcohol by adolescents [24]. At the same time, different from traditional media information, the socialization factors in SNSs reinforce adolescents’ cognition and behavior toward tobacco and alcohol use. Specifically, extra information such as likes and comments on SNSs will provide adolescents with social reinforcement that tobacco and alcohol use can gain support from others [33]. The theory of interpretive level points out that individuals will process information with a low interpretive level for psychologically close information [59]. Compared with traditional media, the influence of peers and the mode of likes and comments on SNSs have narrowed the psychological distance between individuals and tobacco and alcohol information [59]. Close psychological distance leads individuals to make a superficial interpretation of tobacco and alcohol information (such as interpreting tobacco and alcohol use as effective in reducing anxiety and stress or promoting social interaction based on tobacco and alcohol use information and pictures [25]), and the resulting tobacco and alcohol use. Previous empirical studies have shown that watching likes, comments, and other information on drug or alcohol abuse posts on SNSs significantly reduces the activities of those brain regions responsible for self-control and response inhibition, and increase the possibility of risk behaviors [60]. Additionally, even the indirect autopoietic media effect argues that adolescents’ likes and comments on posts related to tobacco and alcohol use can backfire and influence adolescent tobacco and alcohol use [61]. Thus, deviant peer affiliation can act on adolescents’ tobacco and alcohol use through the mediating effect of SNS tobacco and alcohol information exposure.

### 4.3. Digital Literacy as a Moderator

The results also examined the potential individual differences and protective mechanisms in these relationships and found that digital literacy could mitigate the relationship between SNS tobacco and alcohol information exposure and adolescent tobacco and alcohol use. Consistent with previous studies [19,50], not all individuals will have risk behaviors after exposure to bad social media information, in that digital literacy plays an important role in the interpretation and response of bad information on SNSs. Specifically, adolescents with high digital literacy tend to have a higher critical capacity for information and think about its nature [19,20]. Some examples include critically examining whether tobacco and alcohol use will reduce anxiety, thinking deeply about the profit purpose behind tobacco and alcohol advertising, etc. At the same time, adolescents with high digital literacy have higher information-seeking abilities [62] and may test the authenticity of information and obtain scientific answers by searching [63]. For example, searching whether smoking e-cigarettes is harmful to health and whether drinking small amounts of alcohol is beneficial to health. As a result, adolescents with high digital literacy will interpret SNS tobacco and alcohol information more negatively and avoid tobacco and alcohol use more [45]. By contrast, adolescents with low digital literacy levels have a limited ability to criticize and search SNS information, which makes it difficult to avoid the negative impact of SNS tobacco and alcohol information [18].

## 5. Conclusions, Implications, and Limitations

### 5.1. Conclusions

Tobacco and alcohol use among adolescents remains a prominent problem, and researchers have been exploring the factors associated with adolescent tobacco and alcohol use. Based on the ecological techno-subsystem theory, this study attempted to fill in the research gap in the relationship between adolescent tobacco and alcohol use from the perspective of their offline (deviant peer affiliation) and online (SNS tobacco and alcohol information exposure) interaction. Meanwhile, the protective factors of digital literacy in the current information society were explored. The results found that deviant peer affiliation was not only significantly positively associated with adolescent tobacco and alcohol use but also related to the mediating effect of SNS tobacco and alcohol information exposure. Moreover, digital literacy moderated the relationship between SNS information exposure and tobacco and alcohol use. Specifically, the association between SNS tobacco and alcohol information exposure and adolescent tobacco and alcohol use was weaker for those with high digital literacy. 

### 5.2. Theoretical Implications and Practical Implications

This study has some implications. Theoretically, first of all, this study provides insight into the underlying mechanisms of adolescent tobacco and alcohol use from both online and offline perspectives, extending previous research on the effects of deviant peer affiliation on adolescent tobacco and alcohol use. Specifically, deviant peer affiliation was associated with tobacco and alcohol use by influencing SNS tobacco and alcohol information exposure. Second, the cultivation theory was extended by the finding that SNS tobacco and alcohol messages are significantly associated with adolescent tobacco and alcohol use [35]. Previous empirical studies of the cultivation theory have focused on the effects of traditional media on individuals or on the areas of violent behavior and restrictive eating [20,35]. This study provides empirical support for the cultivation theory in the area of SNSs and tobacco and alcohol use. Third, this study started with the ecosystem represented by peers and the technology system represented by SNSs, which enriched the theory of ecological technology microsystems [17]. The research results showed that in the information society, online and offline communication are inseparable. At the same time, offline and online communication jointly affect the behavior of adolescents. Moreover, our study found a buffering protective effect of digital literacy on SNS information and risky behavior. This has important implications for understanding when SNSs influence alcohol and tobacco use behavior among adolescents.

Practically, deviant peer affiliation and SNS tobacco and alcohol information are risk factors for adolescents’ tobacco and alcohol use. In terms of peer affiliation, parents should always pay attention to adolescents’ choice of affiliation objects and advocate for the integration of adolescents into a positive and healthy youth group. In terms of SNSs, relevant departments should strengthen the supervision of tobacco and alcohol advertisements and information. Adults should also be aware of the impact on adolescents or emphasize the health risks of tobacco and alcohol use when posting information about tobacco and alcohol use. Most importantly, parents and schools need to strengthen digital literacy training for adolescents and encourage them to critically view the tobacco and alcohol information on SNSs, such as thinking about the motivation of the publisher of tobacco and alcohol information, recognizing the disadvantages of short-term enjoyment of tobacco and alcohol, etc.

### 5.3. Limitations and Future Research

Several limitations to this study should be noted. Firstly, the cross-sectional nature of the current investigation prevents us from drawing strong causal conclusions. A longitudinal study design or behavioral experimental design needs to be developed in the future to better understand the effect of SNS tobacco and alcohol information exposure. In the meantime, future digital literacy intervention studies should be investigated. The moderating effect of digital literacy was tested by comparing the effect of SNS tobacco and alcohol information exposure on tobacco and alcohol use before and after the intervention. Secondly, a limitation of the study was that the data were retrieved from self-report questionnaires from adolescents. We cannot exclude the possibility of the subjects’ social approvability and inauthentic reporting, as with a large number of previous self-report studies, despite explaining to them the study’s confidentiality and anonymity prior to the actual test. In future research, it might be possible to reduce the impact of this limitation by investigating other subjects’ reports on adolescent behavior. Thirdly, although 725 adolescents from different schools in China were surveyed in this study, the generalization of the results of this study is still limited by the representativeness of the sample. Attention should be paid to the generalization of the findings of this study across different groups. Unlike other age groups (e.g., elementary students and adults), adolescents are more influenced by their peers. In addition, limited by research resources, this study was conducted in a Chinese cultural context, and attention should be paid to extending research findings in a different cultural context. Therefore, it is necessary to explore the mechanisms influencing youth tobacco and alcohol use in different cultural contexts. Specifically, an important factor related to the use of alcohol and tobacco is whether the underlying intention is to fit in with the collective in a collectivist culture or to self-express in an individualist culture.

## Figures and Tables

**Table 1:** Descriptive statistics and correlation analysis of variables.

	*M* (*SD*)	1	2	3	4
1. Deviant peer affiliation	1.640 (0.595)	1			
2. Tobacco and alcohol use	1.188 (0.513)	0.266 ***	1		
3. SNS tobacco and alcohol information exposure	1.759 (0.769)	0.338 ***	0.204 ***	1	
4. Digital literacy	3.868 (0.543)	−0.037	−0.097 **	0.021	1

** *p* < 0.01; *** *p* < 0.001.

**Table 2:** Testing the moderated mediation model.

Regression Equation		Fitting Index			Significance of Coefficients			
Outcome	Predictors	*R*	*R^2^*	*F*	*B*	*t*	LLCI	ULCI
STAIE	gender	0.399	0.159	45.378 ***	0.069	1.285	−0.037	0.175
	age				0.101	6.237 ***	0.069	0.133
	DPA				0.464	10.299 ***	0.376	0.552
TAU	gender	0.427	0.182	26.627 ***	−0.158	−4.728 ***	−0.223	−0.092
	age				0.041	3.939 ***	0.020	0.061
	DPA				0.187	6.270 ***	0.129	0.246
	STAIE				0.080	3.486 **	0.035	0.126
	DL				−0.113	−3.749 ***	−0.172	−0.054
	ESM× DL				−0.119	−3.238 **	−0.191	−0.047

** *p* < 0.01; *** *p* < 0.001; STAIE = SNS tobacco and alcohol information exposure; TAU = tobacco and alcohol use; DPA = deviant peer affiliation; DL = digital literacy.

## Data Availability

The data of this study are available from the corresponding author upon reasonable request.
